# The karyotype of *Aegilops geniculata* and its use to identify both addition and substitution lines of wheat

**DOI:** 10.1186/s13039-019-0428-2

**Published:** 2019-04-02

**Authors:** Yingjin Yi, Ke Zheng, Shunzong Ning, Laibin Zhao, Kai Xu, Ming Hao, Lianquan Zhang, Zhongwei Yuan, Dengcai Liu

**Affiliations:** Triticeae Research Institute, Sichuan Agricultural University, Chengdu, 611130 Sichuan China

**Keywords:** Substitution line, Addition line, Fluorescence in situ hybridization, Single nucleotide polymorphism

## Abstract

**Background:**

The annual allotetraploid species *Aegilops geniculata* harbors a number of traits relevant for wheat improvement. An effective cytogenetic method has yet to be developed to distinguish between each of its 14 chromosomes.

**Results:**

A fluorescence in situ hybridization (FISH) approach was adopted to describe the karyotype of *Ae. geniculata.* Each of its 14 chromosomes was unequivocally recognized using a cocktail of three probes, namely pTa-713, (AAC)_5_ and pTa71. FISH karyotyping was then used to detect and characterize selections from an *Ae. geniculata* × bread wheat wide cross of a chromosome 1M^g^ disomic addition line and three 4M^g^(4B) substitution lines. The identity of the addition line was confirmed by the presence of *Glu-M1*, detected both using an SDS-PAGE separation of endosperm proteins and by applying a PCR assay directed at the *Glu-M1* locus. The status of the substitution lines was validated by genotyping using a wheat single nucleotide polymorphism chip.

**Conclusion:**

FISH karyotyping based on pTa-713, (AAC)_5_ and pTa71 will be useful for determining the contribution of *Ae. geniculata* to derivatives of an *Ae. geniculata* × wheat wide cross. SNP chip-based genotyping is effective for confirming the status of whole chromosome wheat/alien substitution lines.

**Electronic supplementary material:**

The online version of this article (10.1186/s13039-019-0428-2) contains supplementary material, which is available to authorized users.

## Background

*Aegilops geniculata* Roth (syn. *Ae. ovata* L, 2n = 4x = 28, genome formula U^g^M^g^) represents a source of potentially useful genetic variation of relevance to bread wheat improvement. The species is thought to represent a natural allotetraploid between the diploid *Ae. umbellulata* Zhuk. (U genome carrier) and *Ae. comosa* Sibth. et Sm. (M genome carrier) [1]. A number of genes conferring resistance to various diseases have been transferred from this species into bread wheat, notably *Yr40* (resistance against stripe rust), *Lr57* (leaf rust) [2], *Sr53* (stem rust) [3] and *Pm29* (powdery mildew) [4, 5]. Some accessions of *Ae. geniculata* have displayed high levels of water use efficiency [6], and the species overall exhibit a higher tolerance to moisture stress than any of the related species *Ae. markgrafii* (Greuter) K. Hammer, *Ae. longissima* Schweinf. & Muschl., *Ae. searsii* Feldman et Kislev ex K. Hammer or *Ae. speltoides* Tausch [7]. It also carries alleles at the genes encoding endosperm proteins which have been predicted to improve the end-use quality of bread wheat [8, 9].

Genomic in situ hybridization (GISH) has been a very successful technique for discriminating between the chromosomes belonging to the various genomes represented in *Triticeae* species, while fluorescence in situ hybridization (FISH) tends to be used for identifying individual chromosomes. A FISH-based karyotype of *Ae. geniculata* has been established, employing the probe combination pSc119.2, Afa family repeats, pAs1 and pTa71 [10–12]. However, some segments of the *Ae. geniculata* genome lack any probe hybridization sites, meaning that FISH karyotyping needs to be supported for the identification of non-intact chromosomes by a GISH-based analysis. For example, the sites of pSc119.2 hybridization are concentrated close to the telomeres of most chromosome arms, making it difficult to differentiate between chromosomes 1U^g^, 2U^g^, 3M^g^, and 4M^g^ [10, 12]. Here, the objective was to develop a FISH assay able to unequivocally recognize each of the 14 *Ae. geniculata* chromosomes, and to use this assay to characterize a number of derivatives of an *Ae. geniculata* × wheat wide cross*.*

## Methods

### Plant materials

The following taxa were used in these experiments: *Ae. umbellulata* (2n = 2x = 14, carrier of the U genome) accession AS4, *Ae. comosa* (2n = 2x = 14, carrier of the M genome) accession PI551068, *Ae. geniculata* accession AS6, the bread wheat cultivars Yi-yuan 2 (YY2), Chinese Spring and Chuan-mai 41 (CM41), and ten F_7_ derivatives of a wide cross between *Ae. geniculata* and wheat (pedigree AS6/YY2//YY2/3/CM41).

### GISH and FISH analysis

Cytological preparations were carried out using the methods described by Zhao et al. [13]. For GISH analyses, total genomic DNA was extracted from fresh leaves of *Ae. comosa* and *Ae. geniculata*, and labeled with digoxigenin-11-dUTP (Roche Diagnostics GmbH, Mannheim, Germany) via nick translation to use as the probe; non-labeled total genomic DNA of *Ae. umbellulata* and CM41 were used for blocking. The GISH procedure was based on the protocol described by Hao et al. [14], with the exception that the concentration of the probe DNA was changed to 0.1 μg/μL and that of blocking DNA to 3.5 μg/μL. FISH experiments were conducted based on the methods given by Zhao et al. [15]. The following probes were essayed: Afa family repeats [16], pSc119.2, pTa-535 and pTa71 [17], (AAC)_5_ [18], (CTT)_5_ and pTa-713 [13]. Probes were labeled with either FAM or TAMRA by the TsingKe Biological Technology Company (Chengdu, China). The preparations were stained with DAPI (Vector Laboratories Inc., Burlingame, CA, USA) and the fluorescence signals visualized and captured using an BX-63 epifluorescence microscope equipped with a Photometric SenSys DP70 CCD camera (Olympus, Tokyo, Japan). Raw images were processed using Photoshop v.7.1 (Adobe Systems Inc., San Jose, CA, USA).

### Single nucleotide polymorphism (SNP) genotyping

Genomic DNA was extracted from fresh leaves using a plant genomic DNA kit (Tiangen Biotech, Beijing Co. Ltd., Beijing, China). Chip-based genotyping was carried out using the CapitalBio Wheat 55 K SNP array. (www.capitalbio.com); the SNP loci arrayed on this chip represent a sub-set of the Affymetrix® Axiom® Wheat 660 chip, as selected by the Institute of Crop Science, Chinese Academy of Agricultural Sciences (wheat.pw.usda.gov/ggpages/topics/Wheat660_SNP_array_developed_by_CAAS.pdf). The flanking sequences of each locus were used to map each site onto the bread wheat reference sequence (urgi.versailles.inra.fr/download/iwgsc/IWGSC_RefSeq_Assemblies/v1.0/), by imposing a BLASTN E-value threshold of 10^− 10^, allowing a maximum mismatch of one base. For lines concluded to harbor an *Ae. geniculata* chromosome substituting for a wheat chromosome, the ratio between the observed and expected number of markers on the wheat chromosome in question (4B) was calculated by considering a series of 10 Mb intervals along the chromosome, applying a sliding window of 10 Mb and a step length of 1 Mb. A graphical representation of these ratios was obtained using the R package ggplot2 v.2.2.1 [19]

### High molecular weight glutenin subunit (HMW-GS) analysis

The extraction of protein from single grains and their separation using sodium dodecyl sulfate polyacrylamide gel electrophoresis (SDS-PAGE) followed methods given by Yan et al. [20]. After electrophoresis for 150 min (120 V, 20 mA), the gels were stained with Coomassie Brilliant Blue R-250 staining solution for 1 h, then destained in distilled water. The primer pair 5′-ATGGCTAAGCGGYTRGTCCTCTTTG/5′-CTATCACTGGCTRGCGGACAATGG was used to amplify the coding region of the whole set of *Glu-1* genes [21]. The methods required for PCR amplification, cloning and sequencing followed those given by Guo et al. [21].

### Development of a PCR assay for Glu-M1x

In a search for informative SNPs, a multiple sequence alignment was carried out of the gene sequences encoding the HMW-GS 1Dx2 (Genbank accession KF466259.1), 1Ax (JQ007589.1), 1Bx13 (EF540764.1), 1Ux (KX375406.1) and 1 Mx (KX375404.1), using DNAMAN v7 software [22]. This permitted the design of a primer pair (5′-CGCCCTCGTGGCTCTCACCC/5′-TTTGCTGCTGGTATTGTCCA) which specifically amplified the encoding sequence of 1 Mx subunit. The amplicon was generated by exposing the reaction to an initial denaturation of 94 °C/5 min, followed by 30 cycles of 94 °C/30 s, 63 °C/30 s, 72 °C/40 s; the PCR was completed with a final extension step of 72 °C/10 min.

## Results

### The FISH karyotype of ae. Geniculata

**Fig. 1:**
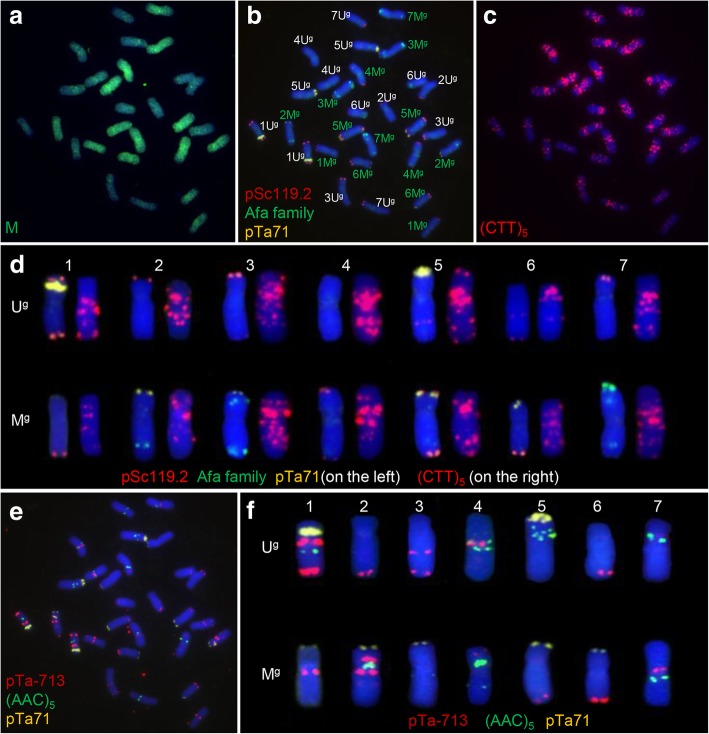
The FISH/GISH karyotype of *Ae. geniculata* accession AS6. **a** GISH differentiates the M genome chromosomes (labeled green) from those of the U genome (blue). **b**, **c** FISH profiling of the mitotic chromosomes of AS6 using as probes (**b**) pSc119.2 (red), Afa family repeats (green) and pTa71 (yellow), (**c**) (CTT)_5_ (red). **d** The FISH karyotype of AS6. The left hand chromosome of each pair shows the hybridization sites of pSc119.2, Afa family repeats and pTa71, while the right hand chromosome of each pair shows the (CTT)_5_ sites. **e** FISH profiling of the mitotic chromosomes of AS6 using as probes pTa-713 (red), (AAC)_5_ (green) and pTa71 (yellow). **f** The FISH karyotype of AS6, based on pTa-713, (AAC)_5_ and pTa71 sites. The images are shown in (a, b, c) and (e) were obtained from a single mitotic cell

When the mitotic chromosomes of *Ae. geniculata* were probed with labeled total genomic DNA of *Ae. comosa* and an excess of unlabeled *Ae. umbellulata* genomic DNA, the M genome chromosomes (labeled green) were readily distinguished from the U genome ones (Fig. 1a). The same preparations were then subjected to FISH using the four probes pSc119.2, Afa family repeats, pTa71 and (CTT)_5_ following suggestions made elsewhere in the literature [10–12, 23, 24]. Most of the pSc119.2 sites were found to lie near the telomeres, while those recognized by the Afa family repeats probe were limited to chromosomes 2M^g^, 3M^g^ and 7M^g^ (Fig. 1b). The FISH profile of AS6 differed somewhat from previously published profiles of the species: for instance, pTa71 sites were located on seven pairs of chromosomes, of which two were on U genome chromosomes (1U^g^, 5U^g^) and the other five on the M genome chromosomes 1M^g^, 2M^g^, 3M^g^, 5M^g^ and 6M^g^ (Fig. 1b). The (CTT)_5_ probe detected sites on the chromosomes belonging to both the U and the M genomes (Fig. 1c). A FISH karyotype of AS6 based on probing with pSc119.2, Afa family repeats, pTa71 and (CTT)_5_ is shown in Fig. 1d. Without the GISH data, it was not easy to differentiate between several of the M and U genome chromosomes: for example, the FISH profiles of chromosomes 3U^g^ and 4M^g^ were almost identical to one another (Fig. 1d). For this reason, experiments were carried out to elaborate a more effective set of FISH probes; these led to the choice of the combination pTa-713, (AAC)_5_ and pTa71. The (ACC)_5_ sites were largely concentrated around the centromeres and the middle of the chromosome arms, while the pTa-713 sites were distributed across several chromosome arms (Fig. 1e, f). The three probes combination allowed for each of the 14 chromosomes of *Ae. geniculata* to be discriminated without the need for an accompanying GISH procedure (Fig. 1f).

### FISH-based identification of introgression materials

**Fig. 2:**
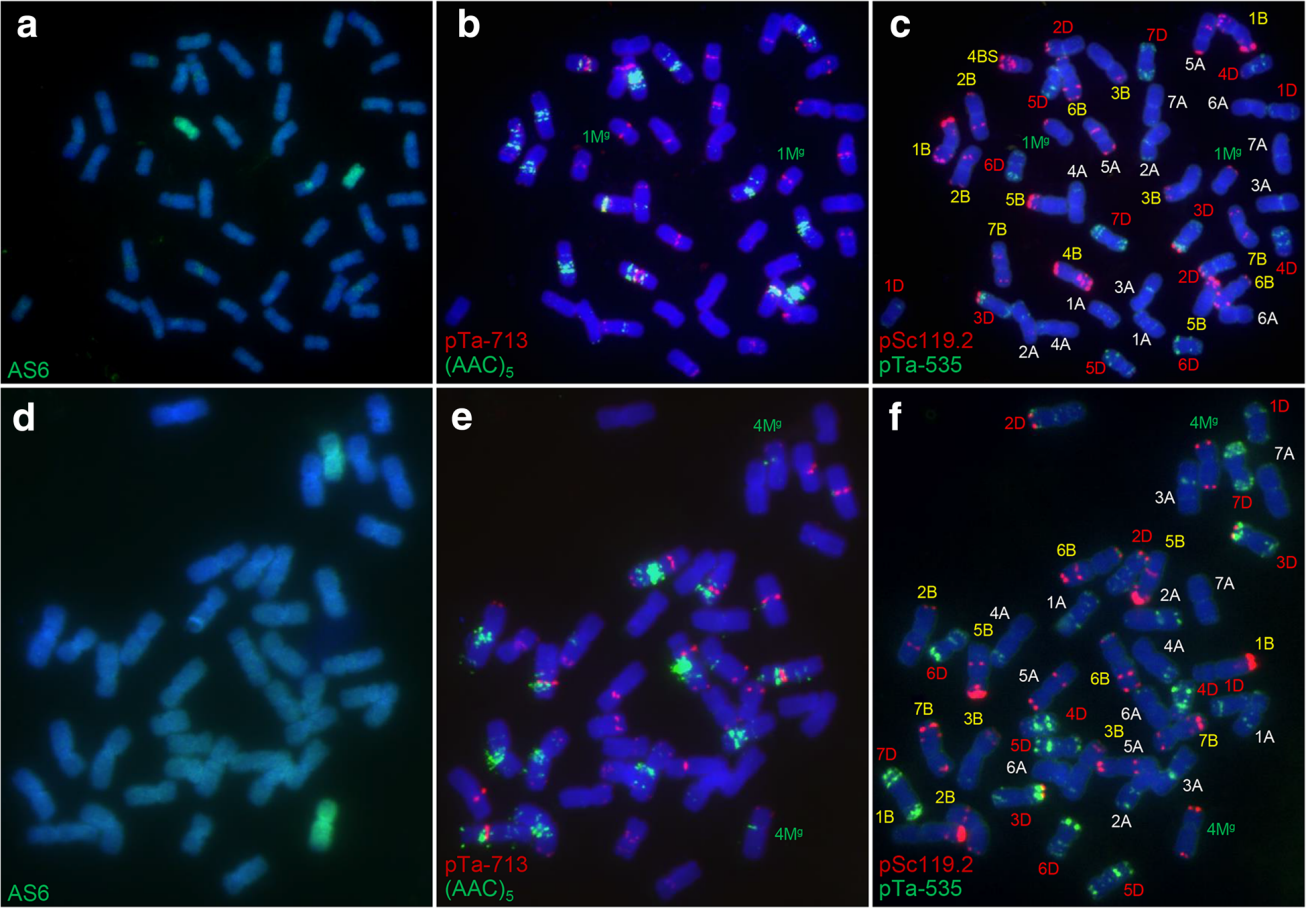
Identification of *Ae. geniculata* chromatin in derivatives of an *Ae. geniculata* × wheat wide cross. **a**-**c** A chromosome 1M^g^ disomic addition line was recognized following (**a**) GISH using labeled AS6 genomic DNA as the probe, (**b**, **c**) FISH with probes (**b**) pTa-713 and (AAC)_5_, (**c**) pSc119.2 and pTa-535. The images shown in (**a**-**c**) were obtained from a single mitotic cell. **d**-**f** A 4M^g^(4B) substitution line was recognized following (**d**) GISH using labeled AS6 genomic DNA as the probe, (**e**, **f**) FISH with probes (**e**) pTa-713 and (AAC)_5_, (**f**) pSc119.2 and pTa-535. The images shown in (**d**-**f**) were obtained from a single mitotic cell

Ten derivatives of a cross between *Ae. geniculata* and bread wheat were subjected to the newly developed FISH assay. One line had a somatic number of 44, while that of the nine others was 42 chromosomes. A GISH analysis demonstrated that two of the chromosomes present in the 2n = 44 line (Add L-1) had been inherited from *Ae. geniculata* (Fig. 2a). Applying the FISH procedure confirmed that these chromosomes comprised a pair of 1M^g^ chromosomes (Fig. 2b, c). Three of the 2n = 42 lines also carried a pair of *Ae. geniculata* chromosomes (Fig. 2d), and the FISH assay showed that all three (Sub L-1, Sub L-2, and Sub L-3) represented a 4M^g^(4B) substitution line (Fig. 2e, f). There was no evidence for the presence of any *Ae. geniculata* chromatin in any of the other six lines.

### The HMW-GS profile of add L-1

**Fig. 3:**
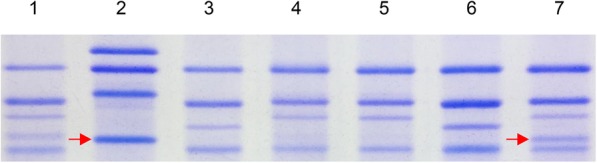
HMW-GS profiling of the endosperm proteins of Add L-1. Lane 1: Chinese Spring, lane 2: AS6, lane 3: YY2, lane 4: CM41, lane 5, 6: an *Ae. geniculata* × wheat derivative lacking *Ae. geniculata* chromatin, lane 7: Add L-1. YY2 and CM41 are part of the pedigree of Add L-1. The arrow indicates the HMW-GS present in the endosperm protein of both Add L-1 and AS6

**Fig. 4:**
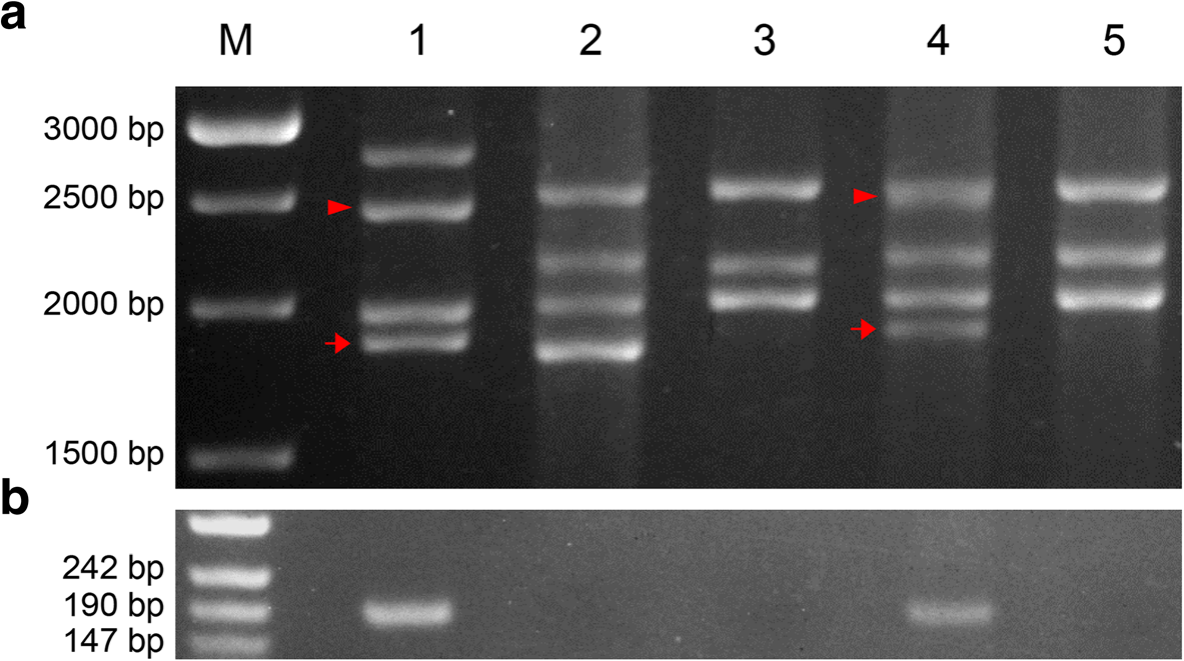
PCR-based genotyping of Add L-1. Lane M: weight marker, lane 1: AS6, lane 2: YY2, lane 3: CM41, lane 4: Add L-1, lane 5: an *Ae. geniculata* × wheat derivative lacking *Ae. geniculata* chromatin. The amplicons were generated by a primer pair targeting (**a**) all *Glu-1* sequences, (**b**) the gene encoding the x subunit of *Glu-M1*. The tailed arrow in (**a**) indicates the fragment amplified from the sequence encoding the Glu-M1y subunit and the tailless arrow from the sequence encoding the Glu-M1x subunit

Since homeologous group 1 chromosomes harbor the *Glu-1* genes which encode HMW-GS, the endosperm protein profile of Add L-1 grain was obtained by SDS-PAGE to identify the presence of the products of the *Ae. geniculata* homeolog of *Glu-1.* The Add L-1 and AS6 profiles both included a subunit not represented in either of the wheat parents of Add L-1 or in any of the other sister lines (Fig. 3). When the *Glu-1* coding region was PCR-amplified from *Ae. geniculata* gDNA, the amplicon was found to include a fragment of the same length as that present in the amplicon generated from an Add L-1 template; the fragment was not represented in amplicons produced from euploid wheat (Fig. 4a). When this fragment was cloned and sequenced, both the Add L-1 and AS6 version proved to be a sequence of length 1860 nt and were of identical sequence. This sequence has been deposited in Genbank under accession number MK135469. The sequence differed from that encoding the 1My subunit (KX375405.1) with respect to seven nucleotides, and their predicted products differed for just one residue. The amplified fragment derived from the gene encoding the AS6 1 Mx subunit was likely too similar to one of the wheat fragments (Fig. 4a) to facilitate its isolation, so a PCR assay was designed to target this gene in order to confirm its presence in Add L-1 (Fig. 4b). The conclusion drawn from these assays was that the *Ae. geniculata* chromosome present in Add L-1 harbored the genes encoding both the x and y subunits of Glu-M1.

### SNP genotyping of the 4M^g^(4B) substitution lines

**Fig. 5:**
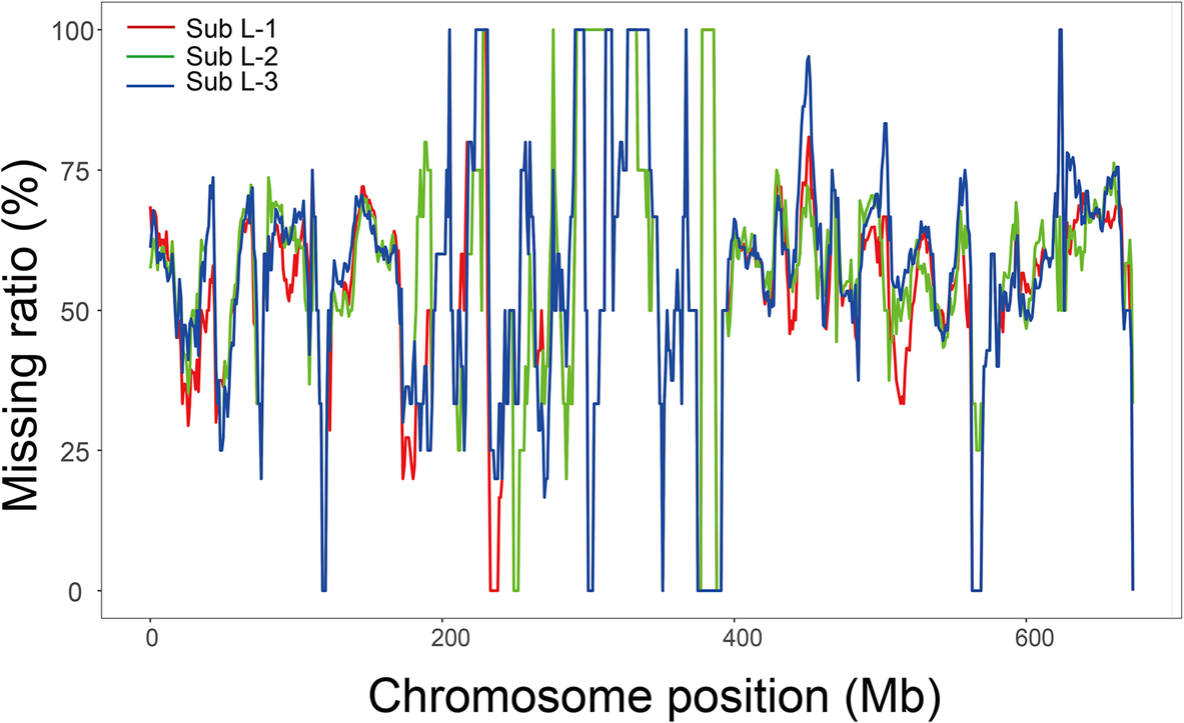
The map location of the 4B SNPs not present in the three 4M^g^(4B) substitution lines Sub L-1 through L-3

The three 4M^g^(4B) substitution lines Sub L-1 through L-3, along with their *Ae. geniculata* parent AS6 and their wheat parents YY2 and CM41 were subjected to SNP genotyping to confirm their FISH-based designation. It was expected that the wheat SNP markers map to 4B (2601) showed a highest ratio to present as missing in the 4M^g^(4B) lines. Thus, the missing markers ratio for individual chromosomes were analyzed. As expected, about 60% SNPs (59.0% for Sub L-1; 60.0% for Sub L-2; 60.6% for Sub L-3) for chromosome 4B that covered the whole chromosome (Fig. 5) showed as missing in the three substitution lines and this ratio greatly exceeded for other chromosomes (Table 1).

**Table 1:** The distribution of missing SNPs on chromosomes in substitution lines

Chromosome	Number	Sub L1		Sub L2		Sub L3	
		Number	Ratio	Number	Ratio	Number	Ratio
1A	2632	35	1.3%	28	1.1%	26	1.0%
1B	2630	27	1.0%	36	1.4%	27	1.0%
1D	2495	19	0.8%	24	1.0%	21	0.8%
2A	2622	14	0.5%	18	0.7%	20	0.8%
2B	2578	33	1.3%	31	1.2%	45	1.7%
2D	2590	204	7.9%	207	8.0%	205	7.9%
3A	2194	31	1.4%	24	1.1%	32	1.5%
3B	2629	32	1.2%	34	1.3%	46	1.7%
3D	2072	17	0.8%	21	1.0%	23	1.1%
4A	2573	34	1.3%	35	1.4%	41	1.6%
4B	2601	1535	59.0%	1560	60.0%	1575	60.6%
4D	1087	28	2.6%	36	3.3%	30	2.8%
5A	2633	37	1.4%	53	2.0%	44	1.7%
5B	2622	39	1.5%	50	1.9%	64	2.4%
5D	2142	33	1.5%	40	1.9%	48	2.2%
6A	2623	40	1.5%	49	1.9%	40	1.5%
6B	2601	94	3.6%	107	4.1%	91	3.5%
6D	2067	30	1.5%	30	1.5%	24	1.2%
7A	2601	28	1.1%	42	1.6%	41	1.6%
7B	2542	212	8.3%	219	8.6%	198	7.8%
7D	2625	23	0.9%	23	0.9%	23	0.9%

Of the 51,159 features represented on the SNP chip, 29,537 (57.7%) also hybridized with a sequence(s) present in AS6. Comparability, about 40% 4B SNPs also present in the three 4M^g^(4B) substitution lines. It suggested a proportion of the SNP assays also recognized a site on 4M^g^. To obtain these SNPs, we filtered 4B SNPs among Sub L-1, Sub L-2, Sub L-3 and AS6. However, ambiguous hybridizing signals may appear using wheat SNP markers to genotype its relative species and introgression lines [25]. Thus, only homozygous SNPs were considered. In all, 240 SNPs covering the whole 4B chromosome presenting in each of the three Sub L lines and AS6, and presenting as same homozygous alleles possibly shared by 4M^g^ and 4B were obtained (Additional file 1: Table S1).

## Discussion

The ability to identify the extent, location and origin of chromatin introgressed into a crop species genome from a species in its tertiary genepool is important for the successful execution of a chromosome engineering experiment. FISH-based karyotyping based on a panel of multi-copy probes can be an effective means of recognizing individual chromosomes [11, 17, 26, 27]. Here, when a set of seven such probes was applied to derive a FISH-based karyotype of *Ae. geniculata*, a combination of just three of them, namely pTa-713, (AAC)_5_ and pTa71, was sufficient to discriminate clearly between each of the species’ 14 chromosomes. The FISH assay could then be applied to detect the origin of *Ae. geniculata* chromatin introgressed into a number of derivatives of a wide cross between this species and wheat, resulting in the recognition of a chromosome 1M^g^ disomic addition line and three 4M^g^(4B) disomic substitution lines.

High density SNP arrays have been shown to be effective for the genotyping of wheat and certain wheat/alien introgression lines [25, 28]. In species containing genomes closely related to those present in wheat, the expectation is that – depending on the extent of the evolutionary separation involved – a proportion of the SNP assays developed within wheat itself will also detect the presence of alien chromatin. Here, around 60% of the wheat SNP loci recognized a site in the AS6 genome, a consequence of the fairly close relationship between *Ae. geniculata* and bread wheat [29–31]. A set of 240 of the 2601 chromosome 4B SNPs behaved in this manner, highlighting loci on both chromosome 4B and chromosome 4M^g^ (Additional file 1: Table S1). The conclusion was that a wheat SNP array can be highly informative for identifying the presence of a wheat/alien chromosome substitution line.

The derivatives of the *Ae. geniculate* × wheat wide cross have inherited their non-nuclear genomes from *Ae. geniculata*, which has resulted in their exhibiting very late maturity [32]. While this trait has complicated the evaluation of their agronomic performance, it is of some interest that the presence of chromosome 4M^g^ induces the formation of supernumerary florets, and also introduces a gametocidal mechanism which generates chromosome breakage in gametes which lack the chromosome [33, 34]. The product of *Glu-M1* has been associated with a positive effect on the rheological strength of doughs made from flour of grains carrying chromosome 1M^g^ [8, 9]. Thus, both the 1M^g^ addition line and the 4M^g^(4B) substitution lines can provide the starting material for producing alien translocation lines of interest to the wheat breeding community.

## Conclusion

This study has established a FISH protocol able to unequivocally identify each of the 14 chromosomes of *Ae. geniculata,* avoiding the need to include a parallel GISH procedure. A wheat SNP array was successfully deployed to confirm the cytogenetic status of three independent 4M^g^(4B) substitution lines. Both the 1M^g^ addition line and the 4M^g^(4B) substitution lines represent materials of potential utility for wheat improvement.

## Additional file

Additional file 1: **Table S1.** The 240 SNP markers shared by Sub L-1, Sub L-2, Sub L-3 and AS6. (XLSX 5838 kb)
